# High level of miR-221/222 confers increased cell invasion and poor prognosis in glioma

**DOI:** 10.1186/1479-5876-10-119

**Published:** 2012-06-08

**Authors:** Chunzhi Zhang, Junxia Zhang, Jianwei Hao, Zhendong Shi, Yingyi Wang, Lei Han, Shizhu Yu, Yongping You, Tao Jiang, Jinhuan Wang, Meili Liu, Peiyu Pu, Chunsheng Kang

**Affiliations:** 1 Department of Radiation Oncology, Tianjin Huanhu hospital, Tianjin, 300060, China; 2 Department of Neurosurgery, Laboratory of Neuro-Oncology, Tianjin Neurological Institute, Tianjin Medical University General Hospital, Tianjin, 300052, China; 3 Key Laboratory of Post-trauma Neuro-repair and Regeneration in Central Nervous System, Ministry of Education, Tianjin Key Laboratory of Injuries, Variations and Regeneration of Nervous System, Tianjin, 300052, China; 4 Department of Neurosurgery, The First Affiliated Hospital of Nanjing Medical University, Nanjing, 210029, China; 5 Department of Neurosurgery, Tiantan Hospital, Capital Medical University, Beijing, 100050, China; 6 Department of Radiology, Tianjin Huanhu Hospital, Tianjin, 300060, China; 7 Department of Neurosurgery, Tianjin Medical University General Hospital, Laboratory of Neuro-Oncology, Tianjin Neurological Institute, Tianjin, 300052, China

**Keywords:** MiRNA, TIMP3, Glioblastoma, Cell invasion, Prognosis

## Abstract

**Background:**

MiR-221 and miR-222 (miR-221/222), upregulated in gliomas, can regulate glioma cell cycle progression and apoptosis, respectively. However, the association of miR-221/222 with glioma cell invasion and survival remains unknown.

**Methods:**

Invasion capability of miR-221/222 was detected by mutiple analyses, including diffusion tensor imaging (DTI), transwell, wound healing and nude mouse tumor xenograft model assay. Further, the target of miR-221/222 was determined by luciferase reporter, western blot and gene rescue assay. The association of miR-221/222 with outcome was examined in fifty glioma patients.

**Results:**

MiR-221/222 expression was significantly increased in high-grade gliomas compared with low-grade gliomas, and positively correlated with the degree of glioma infiltration. Over-expression of miR-221/222 increased cell invasion, whereas knockdown of miR-221/222 decreased cell invasion via modulating the levels of the target, TIMP3. Introduction of a TIMP3 cDNA lacking 3’ UTR abrogated miR-221/222-induced cell invasion. In addition, knockdown of miR-221/222 increased TIMP3 expression and considerably inhibited tumor growth in a xenograft model. Finally, the increased level of miR-221/222 expression in high-grade gliomas confers poorer overall survival.

**Conclusions:**

The present data indicate that miR-221 and miR-222 directly regulate cell invasion by targeting TIMP3 and act as prognostic factors for glioma patients.

## Background

Glioblastoma multiforme (GBM) is the most frequent malignant primary brain tumor in adults. The hallmark of GBM is the widespread diffuse invasion of tumor cells into brain tissues. Owing to the aggressive infiltration into the surrounding normal brain, current therapies fail to control GBM [1,2]. With standard treatment, GBM patients have a median survival of 15.6 months, despite of surgery, radiation therapy and chemotherapy [3]. Thus, there is an urgent need to develop novel therapeutic approaches that focus on controlling the invasion of this malignancy.

MicroRNAs (miRNAs), a recently identified class of endogenous, small (~22 nt), non-protein coding, single-stranded RNA molecules, negatively regulate protein-coding genes by base-pair matching with the 3’ UTRs of mRNAs. MiRNAs are dysregulated in tumors and function as oncogenic miRNAs or as tumor suppressor miRNAs [4]. Growing evidence has indicated important roles for miRNAs in tumor invasion. MiR-21 is an important oncogenic miRNA that promotes cell invasion by regulating multiple genes, including PTEN, RECK and MARCKS in several kinds of cancers, such as glioma, ovarian epithelial carcinoma and prostate cancer [5-7]. In pancreatic cancer cells, miR-146a inhibits the invasive capacity with concomitant down-regulation of EGFR and the NF-kappaB regulatory kinase, interleukin 1 receptor-associated kinase 1 (IRAK-1) [8]. In addition, in glioma cells, miR-7 binds to the EGFR 3’UTR and decreases cell invasiveness by suppressing translation of EGFR [9]. Over-expression of miR-146b inhibits glioma cell invasion by targeting matrix metalloproteinases (MMPs) [10]. However, there is little direct evidence to show the mechanism by which miR-221/222 controls glioma invasion.

In the current study, we demonstrate that high levels of miR-221/222 expression in gliomas confer highly aggressive invasion and poorer overall survival. Furthermore, knockdown of miR-221/222 decreased invasion capability, reduced tumor growth and up-regulated the expression of the target, TIMP3, whereas ectopic expression of miR-221/222 exhibited the opposite effects. These findings indicate that TIMP3 is a critical target of miR-221 and miR-222 and that these two miRNAs could be critical therapeutic targets and survival predictors in glioma.

## Materials and methods

### Tissue samples and clinical data

All patients underwent complete or partial surgical resection at Tianjin Huanhu Hospital and Beijing Tiantan Hospital. Fifty paraffin-embedded glioma specimens with clinical data were collected from January 2006 to June 2006, including 14 grade I-II tumors, 18 grade III tumors and 18 grade IV tumors. Twenty-two frozen glioma specimens, stored in liquid nitrogen, were collected from October 2009 to May 2010, including 9 grade I-II tumors, 6 grade III tumors and 7 grade IV tumors. Paraffin-embedded glioma specimens were used for MiRNA locked nucleic acid (LNA) in situ hybridization and immunohistochemistry, and frozen glioma specimen were used for Real-time PCR. This study was approved by the institutional review boards of the hospitals (the Ethics Committees of Tianjin Medical University, TMUhMEC 2009016) and written informed consent was obtained from all patients. Patients were followed by clinical and laboratory monitoring on a regular basis starting at definitive diagnosis. Disease-specific survival time was defined as the time from definitive diagnosis to disease-specific death.

### MR data acquisition

Patients were scanned with a 3.0 T clinical MR imaging scanner (Magnetom Sonata; Siemens, Erlangen, Germany) equipped with the standard head coil according to a standardized hospital brain tumor imaging protocol. The scans used for this study included diffusion tensor imaging (DTI) and axial contrast-enhanced T1-weighted spin-echo scans. In low-grade gliomas, peritumoral edematous regions were selected by T2 and FLAIR. In every patient, we measure three times in peritumoral edematous different regions with the volume of ROI (5 pixels). DTI data were transferred to a workstation (Inspiron 8200; Dell, Round Rock, TX, USA) for analysis. Data processing was performed using DTIStudio (version 2.4; Johns Hopkins University, Baltimore, MD, USA).

### Cell culture and transfection

Human U251 and LN229 glioblastoma cells, were obtained from the China Academia Sinica Cell Repository, Shanghai, China. The cells were maintained in Dulbecco’s modified Eagle’s medium (Gibco, Los Angeles, CA, USA) supplemented with 10% fetal bovine serum (Gibco), and were incubated at 37°C in a 5% CO_2_ atmosphere. Cell transfection was performed using Lipofectamine 2000 (Invitrogen) according to the manufacturer’s instructions.

### Plasmids and oligonucleotides

2′-OMe-oligonucleotides were chemically synthesized and purified by high-performance liquid chromatography (GenePharma, Shanghai, China). The sequences are: 2'-OMe -miR-221 (miR-221), 5'-AGCUACAUUGUCUGCUGGGUUUC-3'; 2'-OMe -miR-222 (miR-222), 5'-AGCUACAUCUGGCUACUGGGU-3'; 2'-OMe-As-miR-221 (As-miR-221), 5'-AGCUACAUUGUCUGCUGGGUUUC-3'; 2'-OMe-As-miR-222 (As-miR-222), 5'-AGCUACAUCUGGCUACUGGGU-3'. Then As-miR-221 and/or As-miR-222 (200 pmol) were transfected using Lipofectamine 2000 (Invitrogen). Cells transfected with scrambled 2'-OMe oligonucleotides (scramble) were used as control. Wild-type TIMP3 lacking its 3′ UTR in pCDNA3 was provided by Dr. J.Q. Cheng (Molecular Oncology, H. Lee Moffitt Cancer Center, Tampa, FL, USA).

### Real-time PCR

MiRNA expression was analyzed using a Hairpin-itTM miRNA qPCR Quantitation Kit (GenePharma, Shanghai, China) and a DNA Engine Opticon 2 Two-color Real-time PCR Detection System (Bio-Rad, USA), according to the manufacturer’s instructions. Cycle threshold (CT value) was acquired using the software provided by the manufacturer. U6 is used as the normalization gene in Real-time PCR. The real-time PCR data were analyzed using the ddCT method.

### Transwell assay

Cells were detached and resuspended in serum-free medium. Cells (1 × 10^5^ cells/well) were then plated into Matrigel coated invasion chambers (Becton Dickinson) and allowed to invade for 24 hours. The remaining cells in the chambers were removed by cotton swabs and the invading cells on the lower surface of the chambers were fixed with 70% ethanol and then stained with hematoxylin. The number of invading cells was calculated by counting three different fields under a phase-contrast microscope.

### Wound healing assay

Cells were optimized to ensure a homogeneous and viable cell monolayer prior to wounding. Equal numbers of cells were seeded into six-well culture plates. According to the above methods, cells were transfected with As-miR-221/222 or miR-221/222. When the cell confluence reached about 90% at 48 h post-transfection, an artificial homogenous wound was created on the monolayer with a sterile plastic 100 μL micropipette tip. After wounding, the debris was removed by washing the cells with serum-free medium. Migration of cells into the wound was observed after 36 h. Cells that migrated into the wounded area or cells with extended protrusions from the border of the wound were photographed. A total of three areas were selected from each well at random, and three wells of each group were quantified in each experiment.

### Western blot

Parental and transfected cells were washed with pre-chilled phosphate-buffered saline (PBS) three times. The cells were then solubilized in 1% Nonidet P-40 lysis buffer (20 mM Tris, pH 8.0, 137 mM NaCl, 1% Nonidet P-40, 10% glycerol, 1 mM CaCl_2_, 1 mM MgCl_2_, 1 mM phenylmethylsulfonyl fluoride, 1 mM sodium fluoride, 1 mM sodium orthovanadate, and a protease inhibitor mixture). Total protein lysates were separated by SDS-PAGE. The separated proteins were transferred to PVDF membranes. Blots were incubated with MMP2, MMP9 and TIMP3 (Santa Cruz) primary antibodies, followed by incubation with HRP-conjugated secondary antibody. The specific protein was detected using a super signal protein detection kit (Pierce). After washing with stripping buffer, PVDF membranes were reprobed with antibody against β-actin.

### ELISA assay

Twenty-four hours post-transfection, cell culture media were collected and quantified for MMP2 and MMP9 levels using an MMP2 and MMP9-specific ELISA kit (R&D Systems, Minneapolis, MN, USA), according to the manufacturer’s protocol.

### MiRNA locked nucleic acid (LNA) in situ hybridization and immunohistochemistry

Using an antisense locked nucleic acid (LNA/DNA) modified oligonucleotide probe, in situ hybridization was performed with fluorescence in situ hybridization kit (CYBRDI, China). MiR-221 and miR-222-LNA oligonucleotides with digoxigenin modification contained LNAs at five locations (underlined): 5′-GAAACCCAGCAGACAATGTAGCT-3′ (miR-221); 5′-GAGACCCAGTAGCCAGATGTAGCT-3′ (miR-222). LNA-miR-221 uses DIG modification and LNA-miR-222 uses BIO modification in 3' distal end. Sections were deparaffinized and deproteinated, and then prehybridized for 2 h in hybridization liquid in a humidified chamber (50% formamide, 5 x SSC). The probes were added to the sections and incubated overnight at 40°C in a water bath. After washing 3 times, anti-digoxigenin-rhodamine solution was added and incubated for 2 h at room temperature in the dark. Nuclei were counterstained with a DAPI karyotyping kit (Genmed, USA). After washing 3 times, sections were sealed and detected under a fluorescence microscope. In immunohistochemistry assay, sections were incubated with primary antibody (1:100 dilution) overnight at 4°C, then incubated with a biotinylated secondary antibody (1:200 dilution) at room temperature for 1 h, followed by incubation with ABC-peroxidase reagent for an additional 1 h. After washing with Tris-buffer, the sections were stained with DAB (30 mg 3,3 diaminobenzidine dissolved in 100 ml Tris-buffer containing 0.03% H_2_O_2_) for 5 min, rinsed in water and counterstained with hematoxylin. The antibodies used in this study were to MMP2, MMP9 and TIMP3. The quantity and intensity scores were calculated such that a final score of 0–1 indicated negative expression (−), 2–3 indicated weak expression (+), 4–5 indicated moderate expression (++) and 6 indicated strong expression (+++).

### Luciferase reporter assay

The pGL3-WT-TIMP3-3’UTR-Luc reporter was created by the ligation of TIMP3 3’ UTR PCR products into the XbaI site of the pGL3 control vector (Promega, USA). For the reporter assay, cells were cultured in 96-well plates and transfected with pGL3-TIMP3-3’UTR-Luc, and As-miR-221 and/or As-miR-221. Following 48 h incubation, luciferase activity was measured using a dual-luciferase reporter system (Promega).

### Nude mouse tumor xenograft model and treatment

U251 glioma cells were subcutaneously injected into 5-week-old female nude mice (Cancer Institute of the Chinese Academy of Medical Science). When the tumor volume reached 50 mm^3^, the mice were randomly divided into four groups (eight mice per group). Each group was treated with 200 pmol scramble oligo, As-miR-221/222 in 10 μl Lipofectamine, miR-221/222 or PBS through local injection of the xenograft tumor at multiple sites (3–4 injected sites). The treatment was performed once every 3 days for 15 days. On day 28, tumors were harvested, fixed embedded in paraffin. The tumor volume was measured with a caliper twice a week, using the formula: volume = length × width^2^/2.

### Statistical analysis

Descriptive statistics, including mean and ± SE, along with independent sample t-tests, and the Pearson correlation were used to determine significant differences. Kaplan-Meier analysis was employed to assess the survival rate of patients relative to expression levels of miR-221 and miR-222. P < 0.05 was considered a significant difference.

## Results

### Correlation of miR-221/222 expression levels and glioma invasion

**Figure 1:**
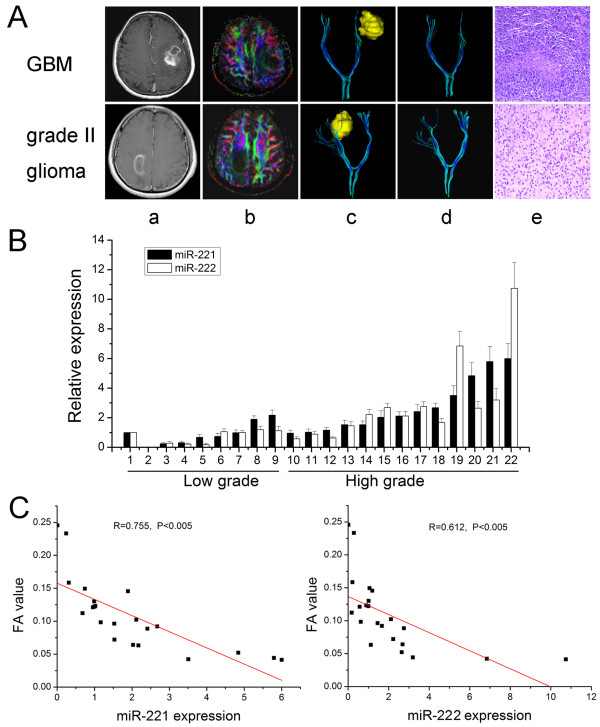
**DTI and expression of miR-221 and miR-222 in gliomas. (A)** DTI in glioma. a: contrast enhanced T1-weighted MRI; b: FA color map; c and d: three-dimensional reconstruction of the fibers surrounding the glioma (yellow); e: HE stain. **(B)** MiR-221 and miR-222 expression in 22 glioma tissues assayed by real-time PCR. **(C)** Correlation between FA values and miR-221 and miR-222 expression in these 22 gliomas by the Pearson correlation analysis.

To evaluate tumor invasion in patients, we employed MR DTI, which is an MRI technique that can indirectly evaluate the integrity of the white matter by measuring water diffusion and its directionality. As shown in Figure 1A, the fractional anisotropy (FA) map and the three-dimensional reconstruction of the fibers surrounding the glioma showed that GBM invaded and destroyed the corticospinal tract, while in grade II glioma the corticospinal tract was pushed around the tumor. For quantitative analysis of miR-221 and miR-222 in frozen tumor tissues of 22 patients, we performed real-time PCR. MiR-221 expression was significantly increased in high-grade gliomas compared with low-grade gliomas, and a similar trend for miR-222 was detected (Figure 1B). Recently, FA values from peritumoral edematous regions have been reported to be a quantitative index of brain invasion by glioma cells and to be negatively correlated with the degree of glioma infiltration [11]. The Pearson correlation showed that a significant negative correlation existed between FA values and miR-221 and miR-222 expression in these 22 gliomas (R = 0.755, P < 0.005 and R = 0.612, P < 0.005, respectively) (Figure 1C). These findings suggest that miR-221/222 may have an important role in glioma invasion.

### Critical role of miR-221/222 in glioma cell invasion

**Figure 2:**
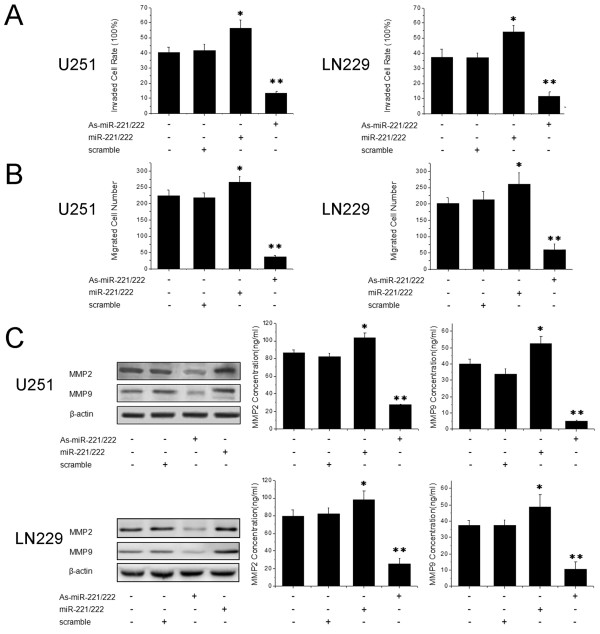
**MiR-221 and miR-222 play an important role in glioma cell invasion. (A)** and **(B)** Cells were transfected with As-miR-221/222 or miR-221/222, and transwell assays and wound healing assays were performed. **(C)** Cells were transfected with As-miR-221/222 or miR-221/222 and MMP2 and MMP9 protein levels were detected by western blot assays. β-actin protein was assayed as a control. MMP2 and MMP9 levels were also measured by ELISA assays. Error bars represent standard deviation and were obtained from three independent experiments. * P <0.05 compared with control group, ** P <0.01 compared with control group.

To further explore the role of miR-221/222 in cell invasion, we performed gain-of-function and loss-of-function analyses by over-expressing or suppressing miR-221/222 with As-miR-221/222 or miR-221/222, respectively. Interestingly, the transwell assay revealed that knockdown of miR-221/222 significantly decreased cell invasion potential compared with cells treated with scrambled oligonucleotide, whereas over-expression of miR-221/222 increased cell invasion (Figure 2A). Consistent with the results of the transwell assay, in the wound healing assay, repression of miR-221/222 significantly inhibited cell migration, while over-expression of miR-221/222 increased migration in both U251 and LN229 cells (Figure 2B). To further explore the proteins relevant to invasion, the level of MMP2 and MMP9 were detected by Western blot and ELISA assays. The expression and secretion of MMP2 and MMP9 were significantly reduced in the As-miR-221/222 group but were up-regulated in the miR-221/222 group (Figure 2C). Therefore, miR-221 and miR-222 are required for glioma cell invasion.

### TIMP3 is a direct target of miR-221 and miR-222

**Figure 3:**
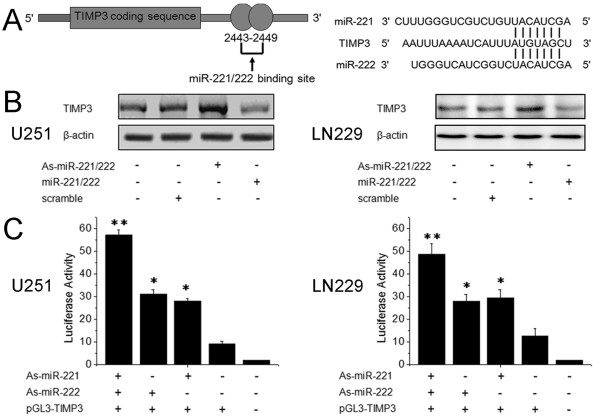
**TIMP3 is a target for miR-221 and miR-222. (A)** Schematic representation of the putative binding sites in the TIMP3 mRNA 3' UTR for miR-221 and miR-222 (the seed sequence (AGCUACAU) was identical, as shown). **(B)** Cells were transfected with As-miR-221/222 or miR-221/222 and TIMP3 protein levels were detected by western blot assays. β-actin protein was assayed as a control. **(C)** pGL3-TIMP3-3'UTR luciferase constructs were transfected into cells that were then treated with As-miR-221 and/or As-miR-222. Luciferase activity was determined 48 h after transfection. The ratio of normalized sensor to control luciferase activity is shown. Error bars represent standard deviation and were obtained from three independent experiments. * P <0.05 compared with control group, ** P <0.01 compared with control group.

To determine the mechanism of action of miR-221 and miR-222 in glioma cell invasion, we performed a miRNA target search using TargetScan and found that the “seed sequence” of miR-221 and miR-222 matched the 3′ UTR of the TIMP3 gene (Figure 3A), which has been evidenced in non small cell lung cancer cells.[12] To detect whether TIMP3 is indeed regulated by miR-221 and miR-222 in glioma cells, we knocked-down miR-221/222 and ectopically expressed miR-221/222 in U251 and LN229 cells. Western blot analysis showed that TIMP3 expression was up-regulated in cells with reduced levels of miR-221/222, whereas TIMP3 expression was down-regulated in cells over-expressing miR-221/222 (Figure 3B). In addition, we created pGL3-WT-TIMP3-3′UTR plasmids. Reporter assays revealed that a reduction of miR-221/222 triggered a marked increase of luciferase activity from pGL3-WT-TIMP3-3′UTR (Figure 3C). These data indicate that miR-221/222 can directly modulate TIMP3 expression by binding to the 3′ UTR of TIMP3 in gliomas.

### Expression of TIMP3 overrides miR-221/222-induced invasion

**Figure 4:**
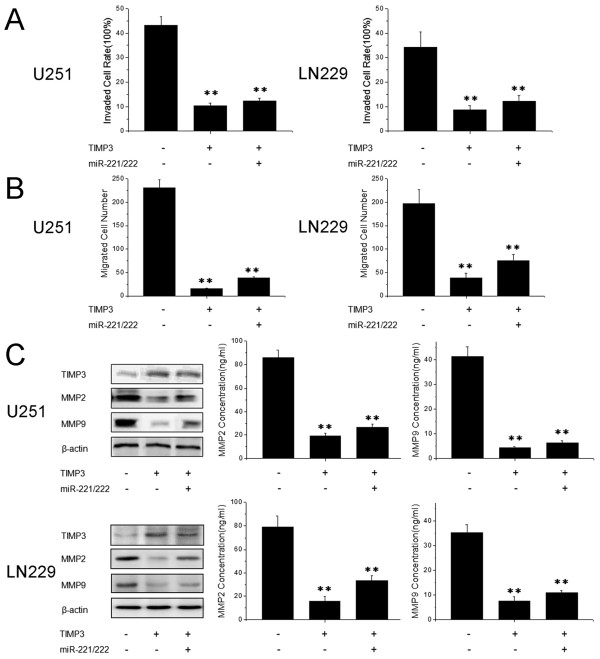
**Expression of TIMP3 abrogates miR-221/222-mediated invasion.(A)** and **(B)** Cells were transfected with pcDNA-TIMP3 and miR-221/222, and transwell assays **(A)** and wound healing assays **(B)** were performed. **(C)** Cells were transfected with pcDNA-TIMP3 lacking the 3’ UTR and miR-221/222 and TIMP3, MMP2 and MMP9 protein levels were detected by western blot assays. β-actin protein was assayed as a control. In addition, the levels of MMP2 and MMP9 were measured by ELISA assays. Error bars represent standard deviation and were obtained from three independent experiments. ** P <0.01 compared with control group.

Having demonstrated TIMP3 to be a direct target of miR-221/222 by our present and other previous studies [12], the importance of TIMP3 in miR-221/222-mediated cell invasion is still unclear. We next introduced TIMP3 lacking its 3′ UTR into U251 and LN229 cells. Transwell and wound healing assays showed that cells transfected with TIMP3 had significantly reduced cell invasion and migration. However, expression of TIMP3 largely abrogated the effects of miR-221/222 on cell invasion (Figure 4A and B). Moreover, similar trends were observed in the expression of proteins relevant to invasion (TIMP3, MMP2 and MMP9) (Figure 4C). These results indicate that TIMP3 is a major target of miR-221 and miR-222 in regulating glioma cell invasion.

### As-miR-221/222 inhibits glioblastoma xenograft growth and induces TIMP3 up-regulation

**Figure 5:**
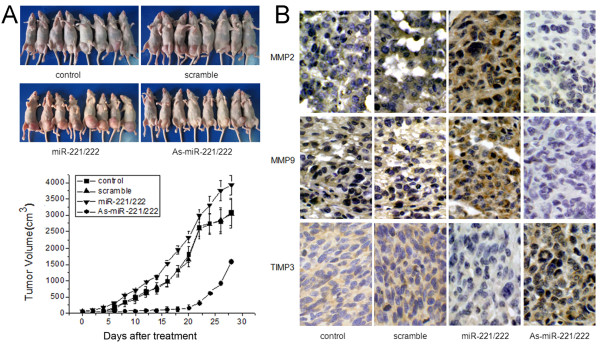
**Effects of miR-221/222 on tumor growth in a xenograft mouse model. (A)** After establishment of subcutaneous tumors, As-miR-221/222 and miR-221/222 were injected in a multi-site injection manner every 3 days for 15 days. Tumor volumes were measured every 2 days during treatment. **(B)** Expression of TIMP3, MMP2 and MMP9 was detected by immunohistochemistry in xenograft tumor sections.

Because the levels of miR-221 and miR-222 are frequently elevated in glioblastoma and because they play an important role in cell survival, we further examined the effects of miR-221/222 on tumor growth in a glioblastoma xenograft model. Reduction of miR-221/222 levels inhibited tumor growth *in vivo*, and over-expression of miR-221/222 slightly increased tumor growth (Figure 5A). Immunohistochemistry then revealed that TIMP3 levels were up-regulated in As-miR-221/222 treated tumors and down-regulated in miR-221/222 treated tumors. In addition, levels of MMP-2 and MMP-9 expression in xenograft tumor sections confirmed the *in vitro* data (Figure 5B). Thus, As-miR-221/222 could be a therapeutic target for glioma intervention.

### Inverse correlation of expression of miR-221/222 and TIMP3 in glioma tissues

**Figure 6:**
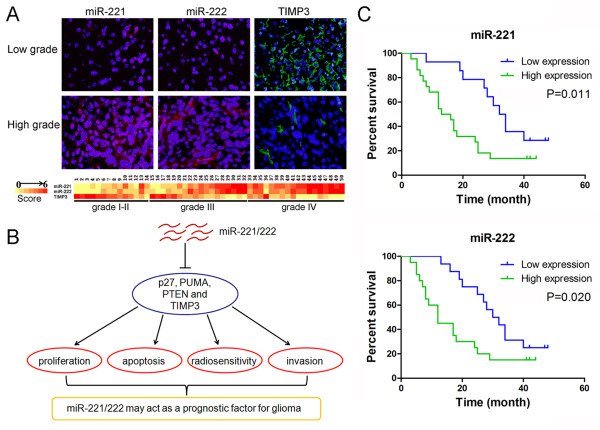
**Clinical significance of miR-221/222 and TIMP3 expression in gliomas. (A)** Expression of miR-221/222 and TIMP3 was analyzed in representative of gliomas with LNA-ISH and immunohistochemical staining. Expression levels of miR-221/222 and TIMP3 were quantified as described in methods. Heat map representing gene expression level is determined in a linear scale with a maximum value of 6 immunoreactive score to highlight differences within this range. **(B)** Schematic representation summarizing data from the present study and earlier reports. MiR-221/222 modulates glioma malignant phenotype by regulating multiple targets, indicating a critical prognostic value for glioma patients. **(C)** Kaplan-Meier survival curves for miR-221 and miR-222 expression. High-grade glioma patients with high levels of miR-221 or miR-222 had a significantly worse outcome. The expression level was categorized as low expression (final score ≤3) and high expression (>3). The number of patients in each group was shown: miR-221, low expression (14 patients) and high expression (22 patients); miR-221, low expression (16 patients) and high expression (20 patients).

Having demonstrated TIMP3 as a major target of miR-221/222, we further investigated the correlation of between miR-221/222 and TIMP3 expression in gliomas. We examined 50 human paraffin-embedded glioma specimens with complete clinical data by miRNA-LNA in situ hybridization. Representative images of miR-221/222 are shown in Figure 6A. According to the expression profile of miR-221/222 and TIMP3, the tissue samples were categorized as low positive (≤3) and high positive (>3). There was an inverse relationship between miR-221/222 and TIMP3 levels in glioma tissues.

### Significant prognostic value of miR-221/222 in high-grade glioma

Our present data and previous studies have shown that miR-221/222 affects the behavior of glioma cells including invasion, proliferation, apoptosis and radioresistance, by regulating multiple targets, including TIMP3, p27, PUMA, and PTEN [13-15]. Thus, we reasoned that miR-221/222 should have a critical prognostic value for glioma patients (Figure 6B). We analyzed the overall survival of patients and found significant differences between the low positive group and the high positive group in high-grade gliomas. Kaplan-Meier survival curve analysis showed that a highly statistically significant correlation was observed between the overall survival and the expression levels of miR-221 (P = 0.011) and miR-222 (P = 0.020) in high-grade gliomas (Figure 6C). These data indicate that the miR-221/222 high positive cases have a markedly worse outcome.

## Discussion

Glioma cell invasion along white matter tracts is believed to be one of the major reasons for the resistance to treatment. A quantitative analysis of glioma cell invasion would be of value for clinical applications and experimental studies. However, in clinical studies, measurements of tumor cell invasion remain controversial. DTI is a new MRI technique that can reveal abnormalities and destruction of white matter fibers and has been used to measure the degree of white matter damage in brain regions, thus providing the potential for quantitative analysis of tumor invasion [16]. Notably, we found that miR-221 and miR-222 are associated with glioma cell invasion by integrating expression and DTI data. In high-grade glioma, DTI indicated a decrease and deflection of the fibers surrounding the glioma that was associated with high levels of miR-221/222. To our knowledge, this is the first report combining analysis of a glioma invasion maker with DTI data.

MiR-221 and miR-222 share the same seed sequence, which are short, evolutionarily conserved regions through which miRNAs bind their target sites in mRNA 3' UTRs, indicating an important role in coordinated regulation and function. Several genes have been found to be common targets of these two miRNAs, such as p27, Bmf and PTEN [13,17,18]. Our recent data have shown that miR-221/222 inhibit cell apoptosis in human glioma cells by targeting the proapoptotic gene, PUMA.[14] Here, we demonstrated that miR-221/222 play an important role in the regulation of glioma invasion by directly targeting TIMP3, an inhibitor of MMPs. Among the MMPs, attention in human gliomas has focused on gelatinases (MMP2 and MMP9) [19]. A major mechanism for controlling the activity of MMPs in the pericellular space is mediated by the action of tissue inhibitors of metalloproteinases (TIMPs), which bind to active MMPs [20]. There are four metalloproteinase family members: TIMP1, TIMP2, TIMP3 and TIMP4. TIMP3 is unique within the TIMP family as it remains closely associated to the extracellular matrix after being secreted by the cell [21]. In our study, down-regulation of TIMP3 correlated with increasing malignancy in human gliomas. TIMP3 was also inversely correlated with miR-221/222 expression. Ectopic expression of TIMP3 inhibited U251 and LN229 glioma cell invasion through the inhibition of MMP2 and MMP9 expression and activity. Furthermore, expression of TIMP3 could largely override miR-221/222-mediated invasion. These results demonstrate that TIMP3 is a core target of miR-221/222 in glioma cell invasion.

The significance of miRNAs in prognostic determination has been shown in a variety of human cancers, such as lung cancer, pancreatic cancer, neuroblastomas and colon cancers [22-25]. However, few studies addressing the correlation between miRNA expression and glioma patient survival have been reported. Zhi et al. screened the expression profile of 200 miRNAs in a sample set consisting of 84 astrocytoma samples and 20 normal adjacent tissue samples and found that low level of miR-181b or miR-106a, or high level of miR-21, was significantly associated with poor patient survival [26]. Another study reported that the cumulative 5 year survival rate of glioma patients was 51.54% in the low miR-182-expression group, whereas it was only 7.23% in the high miR-182-expression group. Moreover, multivariate Cox regression analysis indicated that miR-182 expression was an independent prognostic indicator for the survival of glioma patients [27]. In this study, our data suggest that a high miR-221/222 expression level is a valuable marker for pathological diagnosis and prognosis prediction in high-grade glioma; high miR-221/222 expression levels were significantly associated with poor survival in high-grade glioma patients as determined by Kaplan-Meier analysis. However, recent data showed that miR-221 and miR-222 were downregulated in GBM and neither prognostic nor predictive associations were found for miR-221or miR-222 [28]. Thus, these controversial data needs to be further investigation.

## Conclusions

In summary, our data demonstrate that miR-221/222 regulate glioma cell invasion by directly targeting TIMP3. We also provide direct evidence that high levels of miR-221/222 expression are significantly associated with poorer overall survival. To conclude, our data suggest that miR-221/222 could be intrinsic regulators of progression in glioma cells and could be used as potential targets and predictors of survival in this devastating disease.

## Misc

Chunzhi Zhang, Junxia Zhang, Jianwei Hao and Zhendong Shi contributed equally to this work.

## Competing interest

The authors declare that they have no competing interests.

## Authors' contributions

CZZ and JWH performed the experimental work. JXZ interpreted the data and helped to draft the manuscript. ZDS, YYW, LH and ML participated in the experiments. SZY, YPY, and JHW analyzed data. JT, PYP and CSK conceived of the study and participated in its design and coordination.
